# Standardized Loads Acting in Hip Implants

**DOI:** 10.1371/journal.pone.0155612

**Published:** 2016-05-19

**Authors:** Georg Bergmann, Alwina Bender, Jörn Dymke, Georg Duda, Philipp Damm

**Affiliations:** Julius Wolff Institute, Charité – Universitätsmedizin Berlin, Augustenburger Platz 1, 13353, Berlin, Germany; University of Zaragoza, SPAIN

## Abstract

With the increasing success of hip joint replacements, the average age of patients has decreased, patients have become more active and their expectations of the implant durability have risen. Thus, pre-clinical endurance tests on hip implants require defining realistic in vivo loads from younger and more active patients. These loads require simplifications to be applicable for simulator tests and numerical analyses. Here, the contact forces in the joint were measured with instrumented hip implants in ten subjects during nine of the most physically demanding and frequent activities of daily living. Typical levels and directions of average and high joint loads were extracted from the intra- and inter-individually widely varying individual data. These data can also be used to analyse bone remodelling at the implant-bone interface, evaluate tissue straining in finite element studies or validate analytical loading predictions, among other uses. The current ISO standards for endurance tests of implant stems and necks are based on historic analytical data from the 1970s. Comparisons of these test forces with in vivo loads unveiled that their unidirectional orientations deviate from the time-dependent in vivo directions during walking and most other activities. The ISO force for testing the stem is substantially too low while the ISO force for the neck better matches typical in vivo magnitudes. Because the magnitudes and orientations of peak forces substantially vary among the activities, load scenarios that reflect a collection of time-dependent high forces should be applied rather than using unidirectional forces. Based on data from ten patients, proposals for the most demanding activities, the time courses of the contact forces and the required cycle numbers for testing are given here. Friction moments in the joint were measured in addition to the contact forces. The moment data were also standardized and can be applied to wear tests of the implant. It was shown that friction only very slightly influences the stresses in the implant neck and shaft.

## Introduction

Total joint replacement of the hip is one of the most successful surgical procedures; in Germany alone, it is performed 200,000 times per year. Success rates are still very high, despite the fact that the patients have become substantially younger over the last two decades. In addition, patient expectations after surgery are high; many of them anticipate returning to their full activity level prior to the onset of arthritis and thus challenge the mechanics of the total hip replacement substantially. In the 1970s, many patients were happy to return to walking and stair climbing, while today patients are likely to practice more athletic activities. The currently available data do not reflect the increased activity level of patients, but realistic loads are essential for the pre-clinical assessments that lead to total hip replacement innovations.

### Relevance for a reference load spectrum

Pre-clinical endurance tests must be performed on new implant designs of various implant sizes to prevent mechanical failure, for example, at the taper of modular designs. Finite element studies investigating interface mechanics can enable the development of novel implant geometries and surfaces that improve the adaptation of bone to the implant and prevent loosening, but only if a realistic load spectrum to which the constructs are subjected is known. Friction at the cup, in addition to contact forces, may endanger its fixation stability; this necessitates a more detailed characterisation of the load transfer in the joint. Analytical models of loads in joints, bones and muscles require various model parameters to be defined, but many of them are difficult to identify accurately; thus, the obtained results need to be verified by in vivo data.

Realistic loads can be measured in subjects by using instrumented implants with telemetric data transmission. However, the loads vary within and between subjects and are highly dependent on the kind and level of activity. Rather than using an arbitrary single instance of in vivo data, e.g., from walking, it is therefore favourable to develop a standardized comprehensive reference load *spectrum* for the many conceivable test scenarios, of which only a select few were mentioned above.

### ISO endurance tests on hip implants

Endurance tests of the *stem* are performed according to the ISO7206-4 standard. A sinusoidal force of 2300 N is applied under angles of 10° from medial and 9° from anterior relative to the stem axis. In the following, this test force is referred to as the ‘ISO stem force’. A similar approach is defined in ISO7206-6 for testing the endurance of the *neck*, but the force is increased to 5340 N (‘ISO neck force’). These load directions and magnitudes are based on historic joint contact force calculations [1–3], and a comparison to previously reported in vivo data [4, 5] suggests that the ISO test conditions need to be revised to better reflect the real in vivo loading conditions.

In both ISO tests, 5*10^6^ cycles have to be applied; in practice, 10^7^ cycles are typically exerted. Fatigue of implant metals is determined by the most frequently acting loads, which cause *cumulative* damage over time. Implant metals can withstand static stresses that are approximately 2 to 3 times higher than the fatigue limits [6]. This means that rarely acting extreme forces, such as those during stumbling [7, 8], may cause a sudden fracture of the implant if their amplitudes are 2 to 3 times higher than those during frequent routine activities. A loading frequency between 1 Hz and 30 Hz is specified by the ISO tests. This indicates that the loading rate has no or only a minor influence on the fatigue properties of the stem and neck.

### Previous standardised data on hip contact forces

In 1998, a first set of four patients was assessed, and our team measured telemetrically the hip contact forces and synchronous gait data during some activities of daily living [5]. The complete data were also published on a compact disc [4]. Typical ‘average’ and ‘high’ forces for testing hip implants were determined. However, the results were based on only four subjects, including one subject with an impaired walking ability. Now, an improved instrumented implant [9] enables contact force measurements in more physically capable subjects and additionally allows synchronous measurement of the friction moments in the joint.

The movement data in [4] had been used by [10] to transform the load directions from the femur to the pelvis system. The transformed data and the appropriate loads from an averaged subject [4] are meant to be used for wear tests of hip implants. However, the standardized loads described here are much more representative than the data from 2001. The applicable relative movements between the pelvis and femur will be published in an additional paper.

### Friction moments

In addition to high contact forces, friction moments in the joint may endanger the fixation of acetabular cups, as demonstrated by the frequent implant failures caused by cup loosening [11–13]. For an under-reaming of only 1 mm, a moment of 8.8 Nm was reported to loosen cementless cups after only 5 loading cycles [14], but even values as small as 2.2 Nm have been associated with cup failures [15]. Further statistical analyses suggested that even a more substantial under-reaming of 2 mm, and a moment as little as 3.3 Nm would be sufficient to cause fixation failure in 4.6% of the subjects [16]. Therefore, it is important to compare such stability limits with the real in vivo moments to identify daily activities that may affect *cup* fixation. In addition to the contact forces, friction moments also affect the loads within the *femoral* component. The in vivo friction data allow quantification of this effect and help determine whether the friction moments should be considered when testing neck or stem.

### Goals of this study

Using the in vivo load data captured during demanding and frequent activities of daily living from ten subjects, this study had two main goals:

1. To define reference loading conditions at the hip joint and report typical time courses and peak values of average and high contact forces and moments.
2. To compare the high forces with those from the ISO standards for endurance tests on the neck and stem and, if required, propose more realistic test forces for the ISO standards, based on the new in vivo load spectrum.

## Methods

### Ethics statement

The study was approved by the Charité Ethics committee (EA2/057/09) and registered at the ‘German Clinical Trials Register’ (DRKS00000563). All patients gave written informed consent prior to participation in this study and to have their images published.

### Investigated subjects

Ten subjects participated in this study (Table 1), all of whom suffered from coxarthrosis. Implantations were performed using a standard clinical procedure through a lateral approach. At the time of measurement (Table 1), the participants were in good physical condition and performed all activities without restriction. Only subject H5L had pain at the replaced contralateral hip joint, but with mild pain medication, she was able to perform all exercises without obvious limitations.

**Table 1:** Subject characteristics. Age taken at time of implantation.

Subject		H1L	H2R	H3L	H4L	H5L	H6R	H7R	H8L	H9L	H10R
**Age**	[years]	55	61	59	50	62	68	52	55	54	53
**Sex**	[f / m]	m	m	m	m	f	m	m	m	m	f
**Weight**	[kg]	73	75	92	85	87	84	95	80	118	98
**Height**	[cm]	178	172	168	178	168	176	179	178	181	162

### Measurements

Measurements were taken during the most frequent and highly demanding activities of daily living (Table 2). Based on activity measurements among patients with hip implants [17], these were walking on level ground at a self-selected speed, going up or down a staircase without using the handrail, sitting down or standing up and standing in a one-legged stance. Slow jogging at 7 km/h was additionally studied, as many enjoy this athletic activity and subjects increasingly perform it even after hip replacement [18]. Furthermore, cycling at a moderate power expenditure of 90 W was analysed because it is often practiced by patients with implants as an alternative to walking long distances or as fitness training [19]. Both activities may be performed with a high number of loading cycles and may therefore have a potential influence on implant fatigue.

**Table 2:** Activities.

Activity	Measurement Conditions
**Cycling**	Power = 90 Watts, Speed = 40 rpm, adapted saddle height
**Sit Down / Stand Up**	Without use of arm rest. Seat height = 45 cm
**Knee Bend (Squat)**	Max. knee flexion angles = 51° to 95°, average = 73°
**Walking**	Level walking, speed = 1.0–1.3 m/s; average = 1.1 m/s
**Stance**	Shifting weight from both to one leg and back
**Stairs Up / Stairs Down**	Without use of hand rail. Step height = 19.8 cm, width = 26.3 cm
**Jogging**	7 km/h on treadmill.

All activities, measurement conditions, numbers of analysed trials and postoperative times of measurement are listed in Tables 2 and 3. Most data were captured 10 to 13 months after surgery. For subject H1L, three activities were not measured due to time restrictions of the patient, and no data from jogging were collected for three subjects who were not able to run with confidence at the target speed of 7 km/h. Throughout all activities, synchronously acquired motion capture data were used to determine walking speed and hip flexion angles.

**Table 3:** Postoperative time (in months) of measurement and number of investigated trials. X/Y = X months postoperative / Y trials.

Activity → Subject	Cycling	Sit Down	Stand Up	Knee Bend	Walking	Stance	Stairs Up	Stairs Down	Jogging
**H1L**	13/134	-	-	-	13/31	13/5	13/5	13/4	-
**H2R**	12/42	12/7	12/7	11/8	12/48	12/5	12/7	12/7	15/38
**H3L**	18/25	12/5	12/5	10/4	12/101	12/4	12/8	12/7	-
**H4L**	12/11	3/6	3/6	¾	12/47	12/6	12/6	12/7	12/55
**H5L**	12/40	12/4	12/4	10/7	12/43	12/5	12/7	12/7	19/22
**H6R**	12/17	12/6	12/5	7/5	12/44	12/6	12/12	12/6	12/24
**H7R**	15/81	10/7	10/7	10/9	12/42	12/5	12/14	12/8	12/13
**H8L**	18/32	12/8	12/9	3/5	12/51	12/7	12/16	12/8	18/8
**H9L**	13/21	13/5	13/6	13/3	13/72	13/5	13/7	13/7	13/53
**H10R**	12/29	12/5	12/5	8/8	12/73	12/6	-	-	-

In our subjects, the average resultant contact force during walking became by 1% lower from the first to the twelfth postoperative month [20]. Within the following 21 months (average), it rose by less than 7% (not published). Therefore, the data presented here are assumed to be representative for the whole postoperative time.

### Instrumented implant

**Fig 1:**
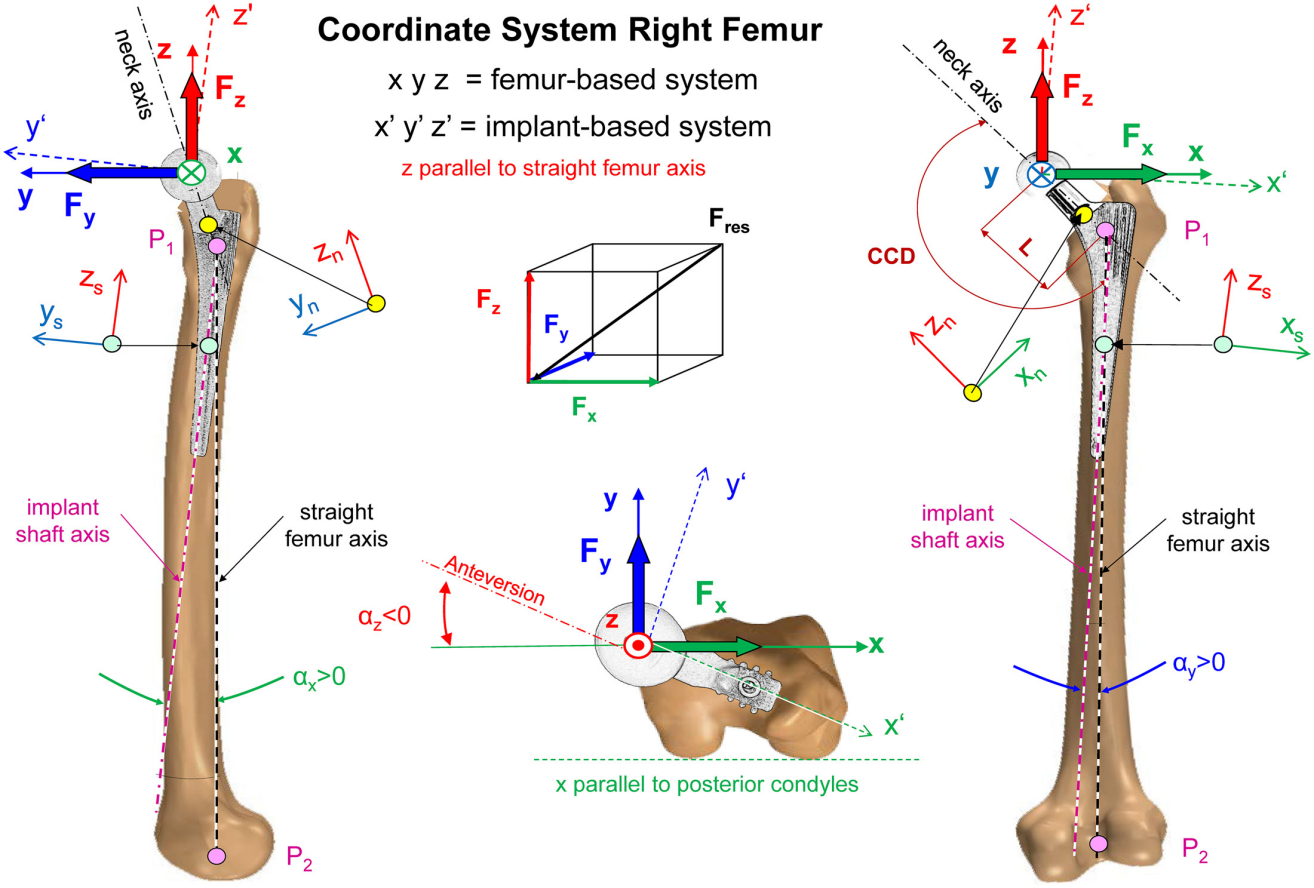
Coordinate system of right femur and implant. x, y, z = axes of femur coordinate system. x = parallel to posterior contour of condyles. P1 = intersection of neck axis and femoral midline. P2 = middle of intercondylar notch. z = straight femur axis between P1 and P2. Force components F_x_, F_y_ and F_z_ act in directions x, y and z. Moment components M_x_, M_y_ and M_z_ turn clockwise around x, y and z. The implant is turned clockwise by angles α_z_, α_y_ and α_x_ around the femur axes z, y and x. α_z_ = anteversion of neck (negative). x’, y’, z’ = axes of implant. x_n_, y_n_, z_n_ = coordinate system at distal end of implant neck. x_s_, y_s_, z_s_ = coordinate system of stem 80 mm below head centre.

A clinically proven titanium implant (CTW, Merete Medical, Berlin, Germany) with a 32 mm ceramic head that articulated with a press-fit cup with XPE inlay was modified to measure the forces and moments acting at the head. The electronics in the hollow implant neck was powered inductively by a coil around the hip joint and was equipped with six strain gauges and a 9-channel amplifier with telemetric data transfer. The electronics were safely encapsulated by welding. This system provided real-time monitoring of the 3D forces and moments with an accuracy of 1–2%. The subject’s activities were video-taped synchronously to the measured load data. The CCD angle of the implants was always 135°; the neck length L was adapted individually (Fig 1, Table 4). The implant, measuring system and calibration are described elsewhere in detail [9, 21, 22].

**Table 4:** Rotation angles for transforming loads from the femur- to the implant-based coordinate system and implant neck lengths. α_x_, α_y_ and α_z_ = orientation of the implant relative to the femur coordinate system in frontal, sagittal and transverse planes (see Fig 1). L = neck length from head centre to intersection of neck and stem axis (see Fig 1). Data from 10 individual subjects and their average.

	Implant Orientation			Neck Length
	Relative to Femur System			
Subject	α_x_ [°]	α_y_ [°]	α_z_ [°]	L [mm]
**H1L**	2.3	-2.3	-15.0	55.6
**H2R**	4.1	0.6	-13.8	59.3
**H3L**	4.0	-3.0	-13.8	55.6
**H4L**	7.5	-1.7	-18.9	63.3
**H5L**	4.0	-2.3	-2.3	55.6
**H6R**	5.8	-1.7	-31.0	55.6
**H7R**	6.3	-1.7	-2.4	63.3
**H8L**	4.6	-1.7	-15.5	59.3
**H9L**	4.6	0.6	-2.3	59.3
**H10R**	1.7	-1.2	-9.7	59.6
**Average**	**4.5**	**-1.4**	**-12.5**	**58.7**

### Measured load components and coordinate systems

The implant measured the three contact force components F_x_, F_y_ and F_z_ in Newton and the three moment components M_x_, M_y_ and M_z_ in Nm (Fig 1). The moments are caused by friction in the joint and act in addition to the forces. The resultant force F_res_ and the resultant friction moment M_res_ were calculated from the vector sums of their components. For the remainder of the study, the term ‘load’ refers to the complete set of all six components and their two resultants, unless otherwise stated. If a force is mentioned without further description, it refers to the peak value during the whole loading cycle.

All loads are reported in the x, y, z coordinate system, which is defined relative to a right-sided *femur* (Fig 1). This is advantageous because then the loads relative to the implant can be recalculated from that data for any prosthesis which is differently oriented relative to the femur, e.g., has a different anteversion. Data from left-sided implants were mirrored to the right side. The origin of this coordinate system is located at the centre of the femoral head. The +z axis points upward and is defined by the line connecting the two points where the curved femoral mid-line intersected with the neck axis (P1) and where it passes the intercondylar notch (P2). The +x axis points laterally and is oriented parallel to the proximal contour of the condyles. The +y axis points in the anterior direction. This definition is in accordance with the ISB recommendations [23] but deviates from our earlier definition [24] for the left hip joint, which defined the +x axis to point medially [4, 5, 8].

### Transformation of loads

To test fatigue of a non-implanted prosthesis, as in the ISO tests, the loads are mostly applied in an *implant*-based coordinate system. To set up such a system, the orientations of all implants relative to the femur were determined from CT images and inter-individually averaged. The implant coordinate system (x_i_, y_i_ and z_i_) is fixed at the implant head but has rotated axes relative to the femur system (Fig 1). It is rotated clockwise by the angles α_z_, α_y_ and α_x_ around the +z, +y and +x axes. The angle α_z_ is the negative anteversion angle of the implant; the other two angles are related to the curvature of the femur in the frontal and sagittal plane. To obtain the force vector F_i_ and the moment vector M_i_ in the *implant* system, the *femur*-based force F and moment M must be multiplied with the transformation matrix T:

$${\text{F}}_{\text{i}}{\text{ = T * F with the force vectors F}}_{\text{i}}\text{ = }\left({\text{F}}_{\text{xi}}{\text{, F}}_{\text{yi}}{\text{, F}}_{\text{zi}}\right)\text{ and F = }\left({\text{F}}_{\text{x}}{\text{, F}}_{\text{y}}{\text{, F}}_{\text{z}}\right)$$ (1)

$${\text{M}}_{\text{i }}{\text{= T * M with the moment vectors M}}_{\text{i}}\text{ = }\left({\text{M}}_{\text{xi}}{\text{, M}}_{\text{yi}}{\text{, M}}_{\text{zi}}\right)\text{ and M = }\left({\text{M}}_{\text{x}}{\text{, M}}_{\text{y}}{\text{, M}}_{\text{z}}\right)$$ (2)

T is given by

$$\underline{T}=\left(\begin{array}{ccc} 1 & 0 & 0\\ 0 & \text{cos}\alpha_{x} & \text{sin}\alpha_{x}\\ 0 & -\text{sin}\alpha_{x} & \text{cos}\alpha_{x} \end{array}\right)*\left(\begin{array}{ccc} \text{cos}\alpha_{y} & 0 & -\text{sin}\alpha_{y}\\ 0 & 1 & 0\\ \text{sin}\alpha_{y} & 0 & \text{cos}\alpha_{y} \end{array}\right)*\left(\begin{array}{ccc} \text{cos}\alpha_{z} & \text{sin}\alpha_{z} & 0\\ -\text{sin}\alpha_{z} & \text{cos}\alpha_{z} & 0\\ 0 & 0 & 1 \end{array}\right)$$ (3)

The implant rotations for the individual subjects and their averages are given in Table 4. The average anteversion was -α_z_ = 12.5°, which was nearly identical to 12.6°, reported as being ‘normal’ (Sutter et al., 2015). To transform the reported loads from the femur to the implant system of our *average* subject, the transformation matrix T takes the following form:

$$\underline{T}=\left(\begin{array}{ccc} 0.9761 & -0.2159 & 0.0251\\ 0.2133 & 0.9738 & 0.0783\\ -0.0414 & -0.0710 & 0.9966 \end{array}\right)$$ (4)

### Calculation of average loads AVER75

The following calculations were performed separately for all activities. First, all measured loads were linearly adjusted from the individual patient’s weight to an average body weight (BW) of 75 kg; this weight approximately corresponds to the average weight of the European population above the age of 60 years (Nordic Council of Ministers, 2011). Then, the time-functions of F_res_ from all trials from the same subject and activity were averaged. The employed ‘time warping’ procedure averaged the cycle durations and then distorted the single trial times such that the summed squared error between all trials and their average was minimized [25]. This algorithm was formulated to best preserve the maxima and minima of the input signals. After morphing the trial times, all patterns of F_res_ were averaged arithmetically. Morphing was based on F_res_ because it contains time-dependent characteristics of all three force components. For each trial the obtained time distortions of F_res_ were then applied to all six corresponding force and moment components to preserve their synchronicity before they were also averaged. In the remainder of this study, forces and moments from *single* subjects always refer to these *individual* averages.

**Fig 2:**
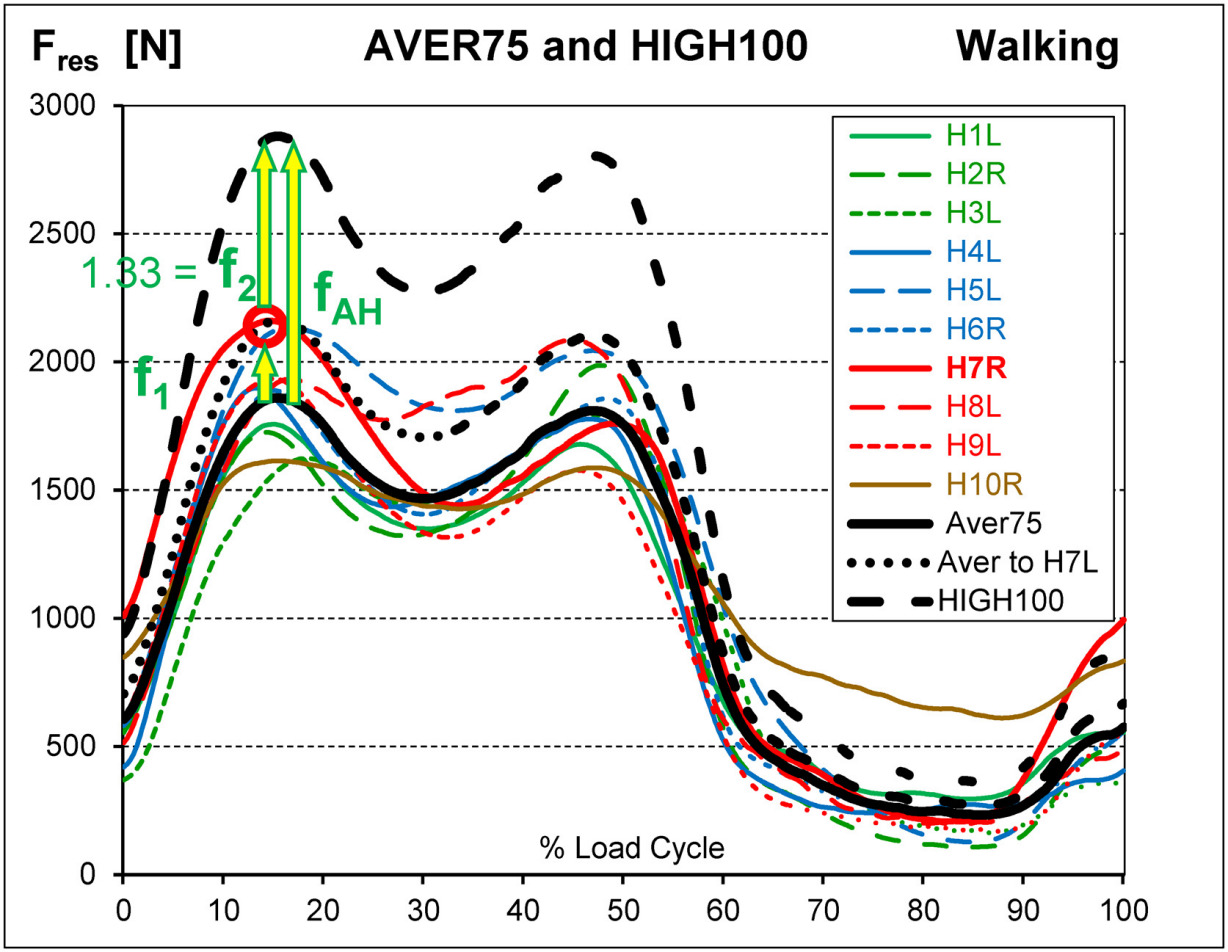
Average force AVER75 and calculation of high loads HIGH100. Resultant forces F_res_ from walking. Coloured lines = forces from 10 subjects with normalized BW = 75 kg. Black solid line = AVER75 force, represents subject of 75 kg with average load levels. Subject H7R had the highest peak value (red circle). AVER75 was multiplied by factor f_1_, to obtain a peak equal to the peak of H7R (black dotted line). The obtained force was further multiplied by f_2_ = 1.33 = 100 kg / 75 kg to obtain the HIGH100 load (black dashed line). The factor f_AH_ = f_1_*f_2_, obtained from the analysis of F_res_, was then applied to all 6 load components. HIGH100 represents subjects of 100 kg with high load levels. For explanation of factors f_1_ and f_2_, please also see Fig 3.

The same procedure was then repeated with the *individual* averages of F_res_ (Fig 2). The resulting six load components were the average loads ‘AVER75’ for an average subject with a BW of 75 kg.

### Calculation of high loads HIGH100

**Fig 3:**
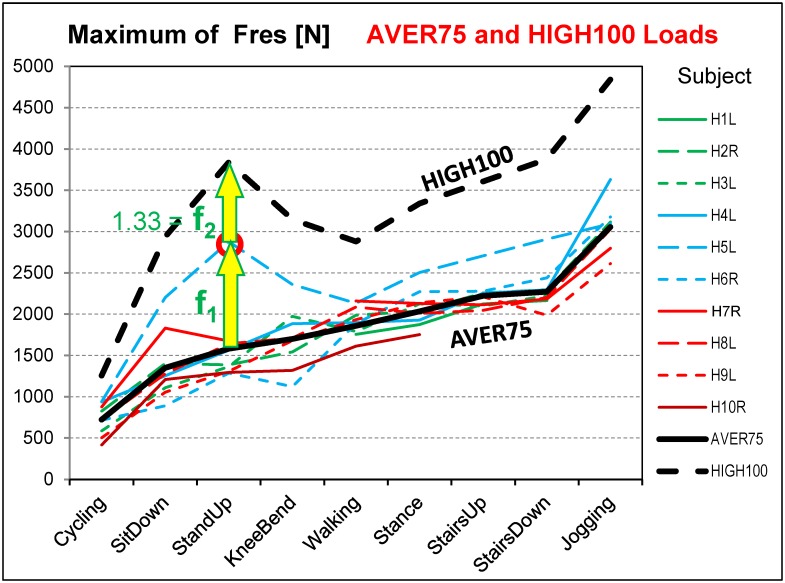
Peak forces F_res_ from nine activities. Coloured lines = individual loads from 10 subjects with normalized BW = 75 kg. Black solid line = average loads AVER75 for BW = 75 kg. Black dashed line = high loads HIGH100 for BW = 100 kg. Factors f_1_ and f_2_ are shown for the data from standing up; for other activities, they are different. See also Fig 2.

Separately for each activity the highest peak value was identified among the individual load-time functions of F_res_ (red circles in Figs 2 and 3). The AVER75 function was then multiplied by a factor, f_1_, which raised its maximum to this peak value. Multiplication by the additional factor f_2_ = 1.33 = 100kg/75kg yielded the high loads ‘HIGH100’. HIGH100 data represent a subject which has the highest peak force relative to all others and a body weight of 100 kg. The resultant multiplication factor f_AH_ = f_1_ * f_2_ (Table 5, bottom), determined from the analysis of F_res_, was then applied to all six load components.

**Table 5:** Magnitudes and directions of AVER75 and HIGH100 peak forces and moments and the conversion factor f_AH_. Average = average of peak force F_res_ from all subjects. Range = range from 10 single subjects. Range [%] = Range in percent of average. α_front_, α_sagit_ and α_trans_ = angles of the peak force vector in frontal, sagittal and transverse planes (see Fig 1). These angles are the same for AVER75 and HIGH100 loads. f_AH_ = factor for calculation of AVER75 from HIGH100 loads for all 6 load components and their resultants: AVER75 = HIGH100 / f_AH_. ^a^/^b^ = ***highest/lowest*** absolute values except from cycling and jogging. * = M_res_ is low at the first peak of F_res_.

Activity → Load Type	Cycling	Sit Down	Stand Up	Knee Bend	Walking	Stance	Stairs Up	Stairs Down	Jogging
**Peak Value of F**_**res**_									
AVER75 Average [N]	731	***1360***^***b***^	1600	1699	1925	2077	2232	***2300***^***a***^	3065
AVER75 Range [N]	416–942	891–2202	1286–2880	1122–2359	1613–2161	1753–2505	2046–2705	1988–2907	2613–3630
AVER75 Range [%]	72	96	100	73	***28*** ^***b***^	36	30	40	33
HIGH100 Average [N]	1256	2935	3839	3145	***2880*** ^***b***^	3340	3606	***3875*** ^***a***^	4839
**M**_**res**_ **at Instant of Peak F**_**res**_									
HIGH100 Average [Nm]	1.11	1.92	***2*.*51***^***a***^	2.49	0.93	1.03	1.93	***0*.*66*** ^b^*	0.80
**Peak Value of M**_**res**_									
HIGH100 Average [Nm]	1.15	2.09	***2*.*77***^***a***^	2.57	1.76	1.72	2.00	2.73*	1.60
**Angle α**_**front**_ **of F**_**res**_ **in Frontal Plane**									
Range [°]	15–27	13–26	16–29	17–28	9–21	8–21	14–25	13–24	13–20
Average [°]	20	20	***24*** ^***a***^	23	17	***16*** ^***b***^	20	***16*** ^***b***^	15
**Angle α**_**sagit**_ **of F**_**res**_ **in Sagittal Plane**									
Range [°]	9–29	2–19	2–14	4–25	0–19	-2–8	10–23	6–18	7–22
Average [°]	17	11	8	8	11	**4** ^**b**^	**18** ^**a**^	11	15
**Angle α**_**trans**_ **of F**_**res**_ **in Transverse Plane**									
Range [°]	22–56	6–53	4–34	7–52	-1–49	-2–34	24–56	19–50	26–61
Average [°]	40	28	17	18	33	***12*** ^***b***^	***42*** ^***a***^	33	44
**Factor f**_**AH**_ [1]	1.73	2.17	***2*.*43*** ^***a***^	1.85	1.55 ^b^	1.64	1.66	1.71	1.59

In the remainder of this study, HIGH100 loads are generally reported, but the spatial directions of the AVER75 forces and moments are identical to those of the HIGH100 loads. The magnitudes and time courses of all AVER75 components and resultants can be obtained by dividing the HIGH100 data by f_AH_. This factor differs between the activities and depends on the inter-subject variability of the F_res_ peak values. The more uniformly an exercise is performed across the cohort, the smaller the difference is between the AVER75 and HIGH100 load magnitudes. This influence of activity is apparent when comparing standing up and walking. Standing up showed larger peak force variations and therefore larger differences between the AVER75 and HIGH100 load magnitudes than walking (Fig 3).

The BW used to calculate the HIGH100 loads was not unrealistically high. The 95% percentile of BW for the average European population from 65–74 years was reported to be 100.5 kg (Nordic Council of Ministers, 2011). Moreover, the 85% percentile of BW for the average male population (70–79 years) in the United States was reported to be 100 kg [26]. If the 95% percentile of BW for the male US population (60–69 years) shall be respected, then even a BW of approximately 125 kg would be realistic, i.e., all HIGH100 should be increased by 25%.

### Friction moments

The friction moments were averaged in the same way as the forces, based on the same time distortions of F_res_. The maxima of the HIGH100 friction moments were compared to published stability limits of cementless acetabular cups to determine whether they were high enough to potentially cause cup loosening and which activities were the most dangerous ones.

### Internal moments in neck and stem when applying ISO or HIGH100 forces

Three exemplary internal moments in the femoral component were calculated for all activities (Fig 1, right side): The bending moment M_bn_ at the distal end of the neck is given by the vector sum of moments M_xn_ around axis x_n_ plus M_yn_ around y_n_. M_bn_ quantifies most of the stress in natural, fracture-stabilized or implant necks and should be generated by the ISO neck test. The torque M_ts_ around the stem axis z_s_ may influence the initial torsional stability of cementless implants in the femur. The total bending moment M_bs_ in the stem is determined 80 mm below the head centre and is most decisive for the stem endurance in the case of a proximally resorbed femur. M_ts_ and M_bs_ should both be realistically generated by the ISO stem test.

The three moments depend not only on the magnitude but also on the spatial direction of F_res_ relative to the implant. M_bs_ is especially sensitive to small directional changes in the three planes (Fig 1) because it depends on the spatial lever arm between F_res_ and the location for which M_bs_ is determined. This dependency on the force directions relative to the femur is equivalent to orientation changes of the implant relative to the bone (α_x_, α_y_, and α_z_; Fig 1). The first two angles depend on the curvature of the femur in two planes, and α_z_ is the anteversion chosen by the surgeon. To quantify this sensitivity, all three angles were modified by ±3° and the largest changes of the three moments during walking were determined, caused by any *combined* angle modification.

### Influence of friction moments on loads in neck and stem

When fatigue testing femoral components, it is unknown whether friction moments can be neglected. To answer this, the three moments in the implant were calculated for all activities under the assumption that only the contact forces were acting at the head. Then, their changes due to the *additional* friction moments were determined. This was performed for the instance of peak F_res_, based on the average geometry and orientation of the implants (Table 4).

### Comparison of ISO test forces with in vivo HIGH100 forces

The magnitude and orientation of the ISO test forces were compared to those of the HIGH100 forces from all activities. Additionally the peak values of M_bn_, M_bs_ and M_ts_ in the implant, which are caused by the ISO forces, were compared with their in vivo values during the various activities. This shows whether the test forces realistically simulate the in vivo loading in the neck and shaft.

## Results

### Peak forces AVER75

The AVER75 peak values of F_res_ varied considerably between subjects (example for walking in Fig 2) and activities (Fig 3; Table 5). AVER75 values during double-legged activities (sitting down, standing up, knee bend) were lower than those during all single-legged exercises, except cycling. Among the single-legged activities, walking caused the lowest forces. The force acting when going down stairs was slightly higher than that during stair climbing.

For the single-legged activities, the ranges of *individual* peak forces were 28%– 40% of the *average* AVER75 values (Table 5). The smallest range (28%) was measured during walking, the most frequent cyclic activity. Cycling had a small average AVER75 value and therefore its individual AVER75 range (73%) was much higher. For the double-legged activities, the range of peak F_res_ was 73%– 100% of the averages, which reflected the large individual variability in left or right support during these exercises.

### Peak forces HIGH100

The HIGH100 peak values of F_res_ consistently exceeded their corresponding AVER75 values by more than a factor f_2_ of 1.33 (Table 5), the expected rise when only increasing the BW from 75kg to 100kg (Figs 2 and 3). Across the different activities, the factor f_AH_ varied between 1.55 and 2.43 and reflected the amount of variation in peak force between subjects. The multiplier f_AH_ was smallest for walking and generally larger for the three double-legged activities. The increased values were caused by the AVER75 forces of subject H5L (the patient with a somewhat painful contralateral hip joint), which were much higher than for all other subjects (Fig 3). As a result, the HIGH100 peak force of 2880 N during walking was the lowest from all daily activities except cycling. When excluding cycling and jogging, the HIGH100 peak forces all laid in a range from 2880 N to 3875 N. The force during jogging (4839 N) was 68% higher than during walking and reached nearly five times the BW.

### Time course of AVER75 forces

The individual magnitude of F_res_ during walking varied substantially, but the general shape of the time courses were very similar across all subjects (Fig 2). When walking, a double-peak pattern was observed for F_res_, with the first maximum, on average, only slightly higher than the second one. The first force peak occurred approximately at the time of contralateral toe-off, while the second peak was associated with the contralateral heel strike. The AVER75 time courses of all six load components during walking and all other activities are identical to those of the HIGH100 patterns if the latter are divided by the activity-specific factor f_AH_ (Table 5).

### Time courses of HIGH100 forces

**Fig 4:**
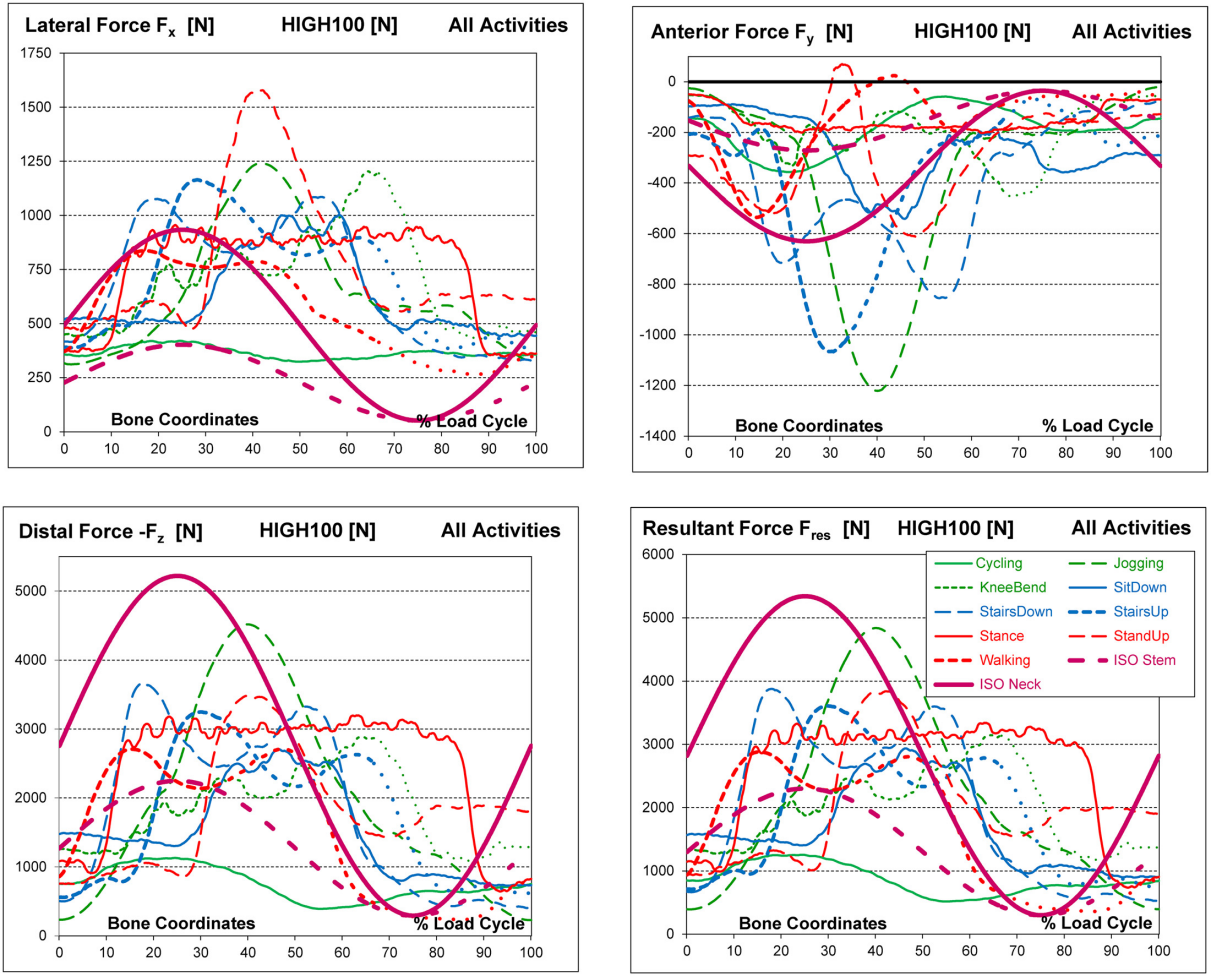
High100 forces during nine activities and ISO forces. High forces for a simulated subject with a body weight of 100 kg. Components F_x_, F_y_ and F_z_ and resultant force F_res_ in the femur coordinate system.

In Fig 4, the three HIGH100 force components and F_res_ during all activities are charted over time; the maxima and minima of the components are listed in Table 6. The extrema of single force *components* did not always occur at the same time as the maximum of F_res_ (Table 5).

**Table 6:** Minima and maxima of HIGH100 force and moment components. Minima/Maxima within whole loading cycles. ^a^/^b^ = ***highest/lowest*** values from all activities except cycling and jogging. All moment components changed their signs within the cycle. See also Fig 4.

Activity → Load Component		Cycling	Sit Down	Stand Up	Knee Bend	Walking	Stance	Stairs Up	Stairs Down	Jogging
**Force**	**F**_**x**_	323/420	442/1002	472/***1578***^***a***^	428/1202	265/***837***^***b***^	347/956	379/1164	329/1085	311/1241
[N]	**F**_**y**_	-357/-58	-541/-88	-612/70	-455/-52	-536/24	-203^b^/-50	***-1067***^***a***^/-68	-855/-78	-1222/-21
	**-F**_**z**_	392/1131	728/***2709***^***b***^	753/3480	1069/2882	235/***2709***^***b***^	644/3198	558/***3876***^***a***^	405/3662	229/4519
**Moment**	**M**_**x**_	-0.60/1.11	-2.03/1.57	***-1*.*11***^***b***^ ***/2*.*61***^***a***^	-1.97/2.39	-1.28/1.47	-1.33/1.41	-1.21/1.82	***-2*.*06***^***a***^ ***/1*.*19***^***b***^	-1.44/0.97
[Nm]	**M**_**y**_	-0.22/0.19	0.00/***0*.*77***^***b***^	-0.70/0.78	0.31/0.93	-0.37/1.21	-0.71/***1*.*71***^***a***^	-0.51/1.24	***-0*.*88***^***a***^ /1.50	-0.49/1.07
	**M**_**z**_	-0.25/0.25	-0.57/0.34	***-0*.*22***^***b***^/0.70	-0.63/0.43	-0.50/0.63	-0.55/***0*.*93***^***a***^	-0.44/0.44	***-1*.*04***^***a***^***/0*.*30***^***b***^	-0.72/0.13

Changes in the forces generally followed smooth patterns, except for the single-legged stance, which required considerable muscle activity to maintain balance. Walking generated the smallest absolute magnitudes of the components F_x_ and F_z_ (Table 6, first and third lines) when cycling was excluded. For F_y_, walking generated the third smallest absolute magnitudes, behind those generated during stance and knee bend.

F_x_ was always positive throughout all activities (Fig 4), indicating that the contact force permanently acted from medial on the joint. The highest value of F_x_ was not found for the activity with the highest F_res_ (jogging) but for standing up, followed by jogging and knee bend (Table 6). It must be noted that HIGH100 loads describe the *average* across subjects. *Individual* data fluctuated around the average numbers. F_y_ was generally negative throughout the whole cycle except during walking and standing up, when *very* small positive values were observed for short periods of time. The joint was therefore nearly always loaded from anterior. When going up stairs, the peaks of F_y_ were close to the magnitudes during jogging. Because the contact force always acts downward, -F_z_ was consistently positive.

**Fig 5:**
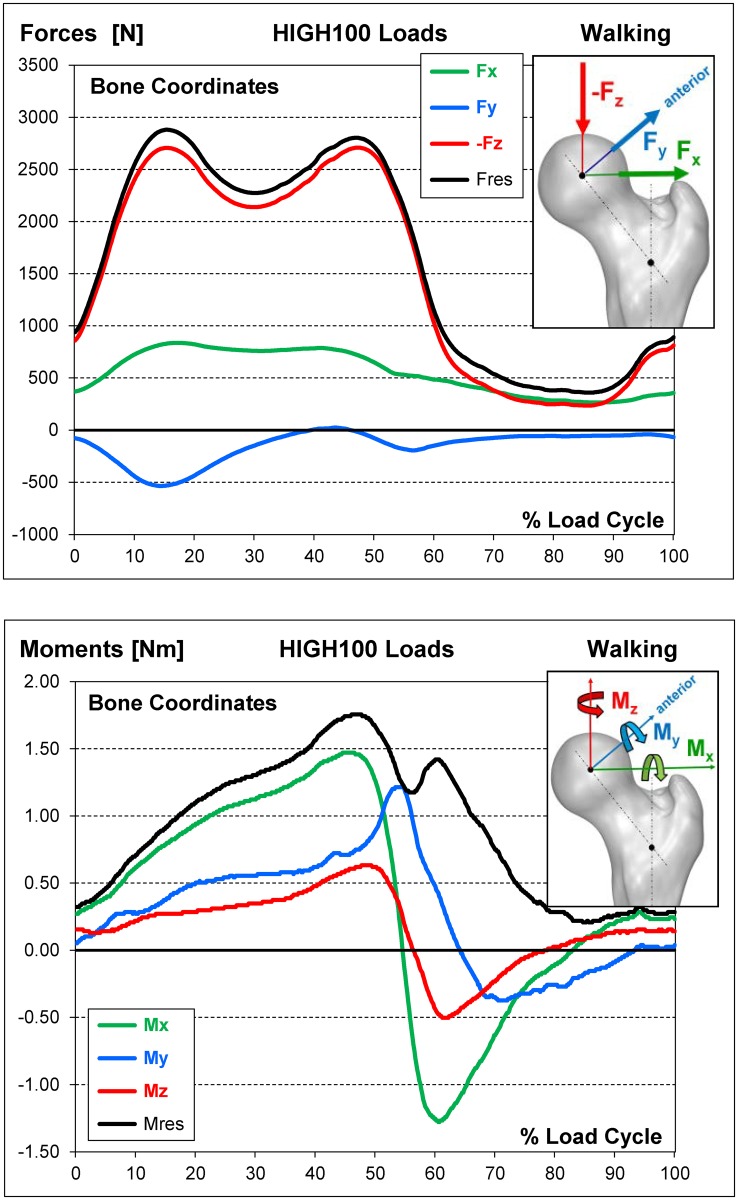
HIGH100 loads during one gait cycle of walking. Top = 3 force components and resultant force. Bottom = 3 moment components and resultant moment. To obtain average loads AVER75 instead of the high loads HIGH100, all components must be divided by the factor f_AH_ (Table 5).

The HIGH100 time course patterns of all load components for walking, the most frequent high-demand daily activity, are combined in Fig 5.

### HIGH100 friction moments

**Fig 6:**
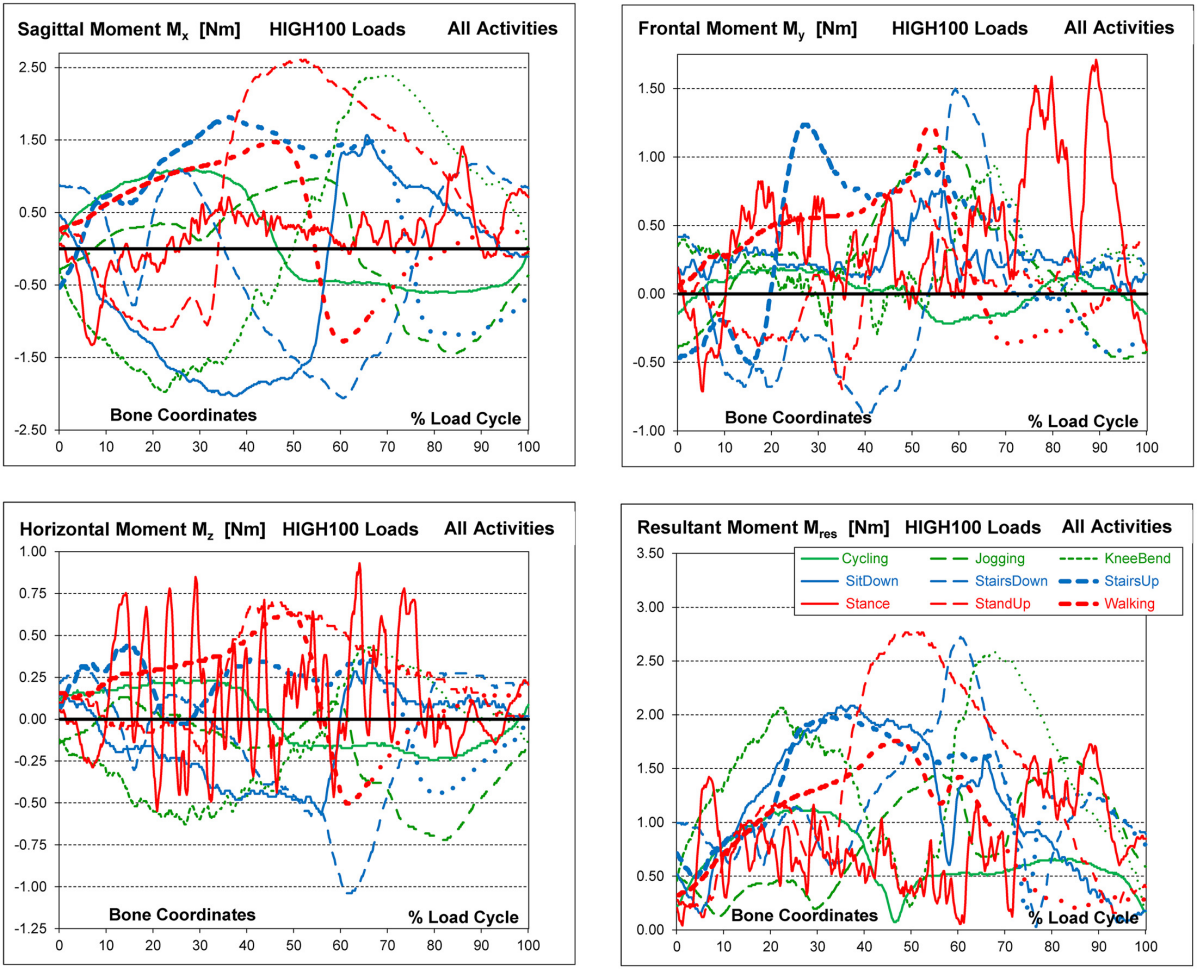
High100 moments during nine activities. High loads for a simulated subject with a body weight of 100 kg. Components F_x_, F_y_ and F_z_ and resultant force F_res_.

The moment components (Fig 6) differed much more between the activities than the force components. All components changed their signs, or at least reached zero, during the loading cycles of all activities (Table 6).

The instability of the single-legged stance was reflected by excessive, random variations of all moment components. The multiple high moment peaks in all three planes did not occur at identical time points. Therefore, these high peak *components* did not result in equally excessive peaks of the *resultant* moment, M_res_.

Positive values of M_x_ are defined as occurring during extension. The highest positive M_x_ values were found for standing up, getting up from knee bend and stair ascending, i.e., for activities involving large extending movements (Table 6, Fig 6). Often, the absolute minima of M_x_ were in the same order of magnitude as the maxima or even larger. During jogging, the maxima of M_x_ were lower than during all other activities and even lower than during cycling. Six activities generated values similar to or higher than the (absolute) M_x_ minimum during jogging. The highest positive values of M_y_ were observed when standing, followed by going down and up a staircase. During several activities, the minima and maxima of M_y_ exceeded those during jogging. The highest negative value of M_z_ occurred when going down stairs; it far exceeded the values during jogging and all other activities. The highest positive value of M_z_ was observed during the single-leg stance.

In contrast to the *force* components, where the axial component–F_z_ dominated the two transverse components and nearly equalled the resultant force F_res_, all three *moment* components had similar magnitudes and equally affected the resultant moment M_res_. The highest peak values of M_res_ were found for standing up, descending stairs and knee bend (Table 5). If cycling was disregarded, jogging caused a lower M_res_ peak than all other activities; here, the moment was 6% or 9% lower than during walking or standing, respectively. M_res_ did not always reach its maximum at the same instant as F_res_ (Fig 6). When going down stairs, M_res_ was very low at that instant (* in Table 5).

### Force directions

**Fig 7:**
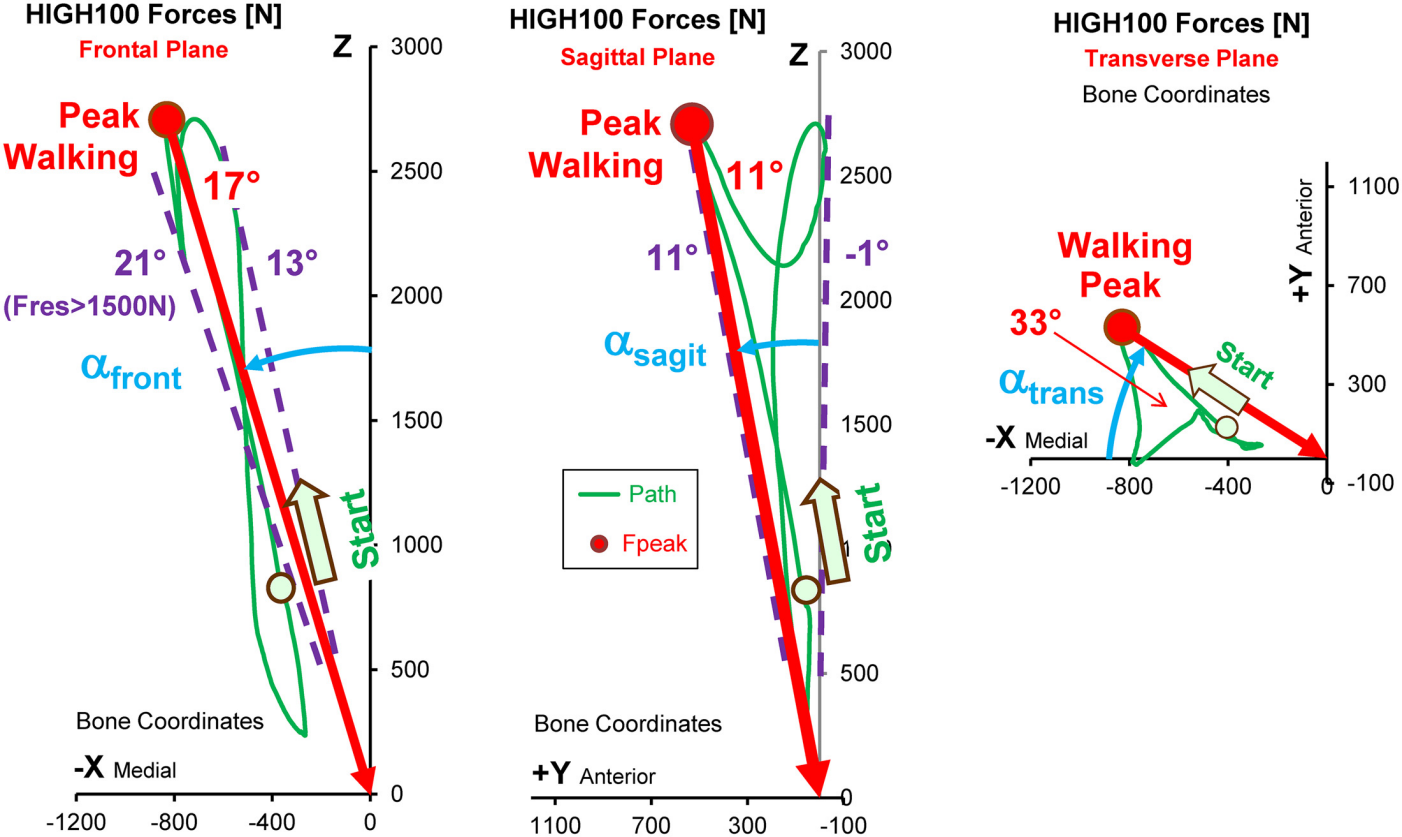
Peak force and path of force vector during one cycle of walking. HIGH100 forces from the average subject. Left = frontal plane. Middle = sagittal plane. Right = transverse plane. The vector acts from the green trajectory towards the head centre. ‘Start’ and circle = point on trajectory at the instant of heel strike. Red dots = directions of the peak force. Dashed lines = limits of force directions throughout the cycle for forces > 1500 N. Angles α_front_, α_sagit_, α_trans_ = angles of the force vector in 3 planes.

During all activities, the directions α_front_ and α_sagit_ of peak F_res_ did not change much *throughout the single loading cycles*. This limited range of load directions was most pronounced in the frontal plane and for high forces. During walking), for instance, high forces above half of their peak value had directions which only varied by 8° in the frontal and 12° in the sagittal plane(dashed lines in Fig 7. The lower the force, the less its direction remained constant.

**Fig 8:**
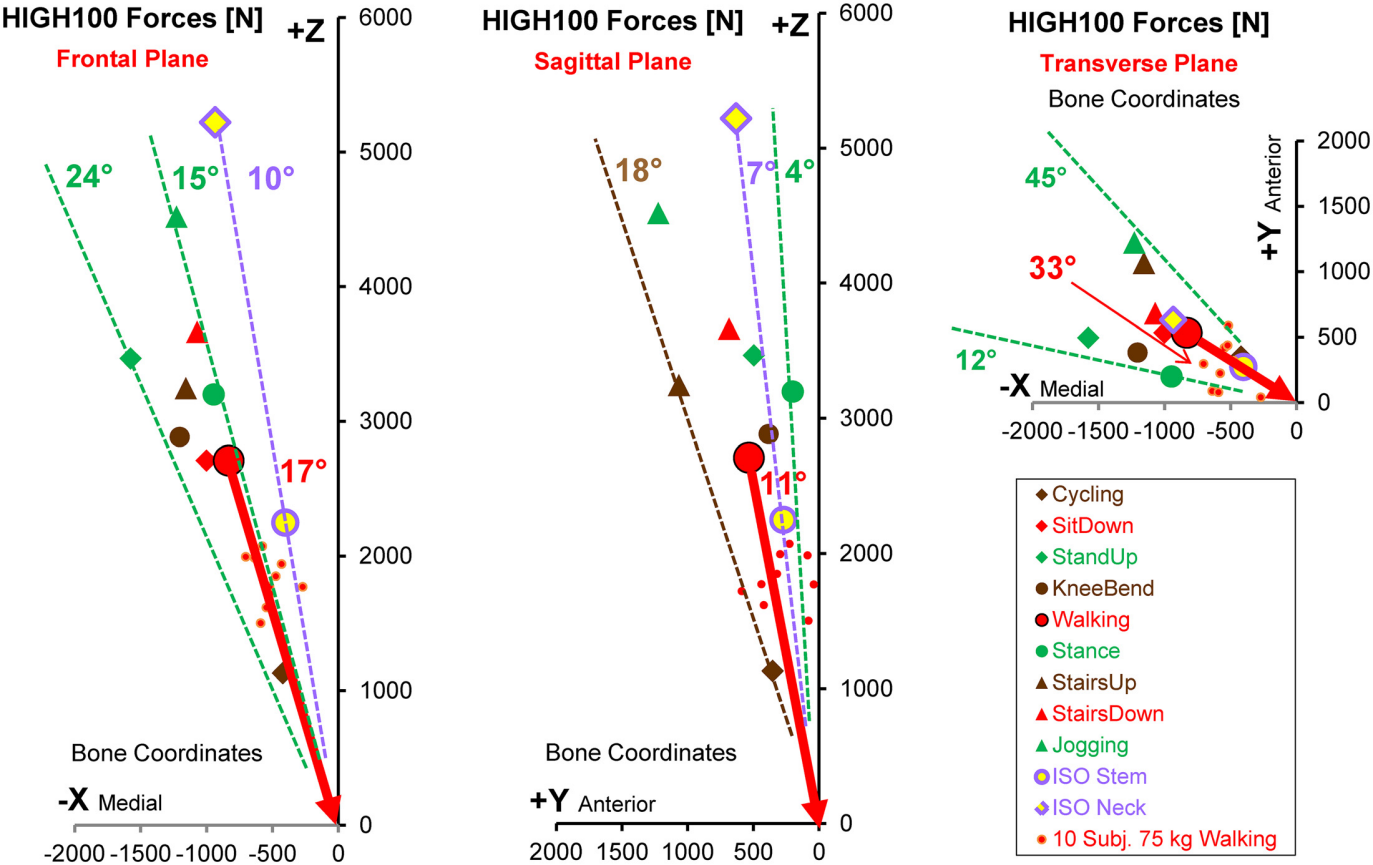
Magnitude and direction of HIGH100 peak forces during 9 activities. High loads HIGH100 from the average subject. Left = frontal plane. Middle = sagittal plane. Right = transverse plane. Vectors of peak forces act from the symbols (different activities) towards the head centre. Red vector = direction during walking. Small red dots = AVER75 peak forces from individual subjects during walking, indicating the inter-individual variation of force directions for the same activity.

Small differences in the load directions were also found when the average peak forces from *different activities* were compared (Fig 8). The load angles varied by only 9° in the frontal and 14° in the sagittal plane. In the transverse plane, the range was considerably larger, reaching a value of 33°.

However, the extent of force direction variation was even larger *between the single subjects* than between different activities. This is illustrated by the small red dots in Fig 8, indicating the *individual* peak force directions during *walking*. For the various activities, the force directions individually varied by 7° to 13° in the frontal and 6° to 21° in the sagittal plane (Table 5).

### Comparison of ISO test forces with in vivo HIGH100 forces

The peak magnitudes of the resultant and all three components of the ISO *stem* force were much smaller than in the HIGH100 data during every activity except cycling (Fig 4). They did not even simulate the magnitudes during walking. In contrast, the *magnitude* of the ISO *neck* force was even larger than the highest in vivo magnitude, found during jogging. However, especially the F_x_ *component* of the ISO neck force was substantially smaller than in vivo. The inadequacy of F_x_, as defined in the ISO tests, was further reflected in the direction of the ISO force in the frontal plane (Fig 8, left). While the ISO test proposes that the force should form an angle of 10° with the z-axis, the in vivo data suggested that this angle should be between 15° and 24° (Table 5). For simulating walking, the most frequent and demanding activity, this angle should have been 17°. In the sagittal plane (Fig 8, middle) the load angle of both ISO forces (7°) fell within the in vivo range determined across all activities (4° to 18°), but was smaller than the in vivo value during walking (11°).

### Internal moments in neck and stem when applying ISO or HIGH100 forces

The internal *torque* M_ts_ = 16.0Nm in the stem (Table 7), caused by the ISO stem force, was only sufficient to simulate the in vivo conditions during cycling, knee bend and stance. However, it was up to 66% smaller than the in vivo magnitudes during walking, ascending or descending stairs. Similar findings were observed for the *bending* moment M_bs_ in the stem, which was 69.6 Nm using the ISO stem force. Although it was large enough to simulate walking, it was substantially lower than the in vivo values when jogging or using a staircase.

**Table 7:** Bending of implant neck and torque around stem. HIGH100 data at the instance of peak force F_res_. M_bn_ = bending moment at base of neck (Fig 1) without friction, only caused by F_res_. M_ts_ = torque around stem without friction. M_bs_ = bending moment in stem without friction. ΔM_bn,_ ΔM_ts_ and ΔM_bs_ = changes due to additionally acting friction moments at head. ISO Neck/Stem = ISO 7206-6/4 test forces.

Activity → Moments	Cycling	Sit Down	Stand Up	Knee Bend	Walking	Stance	Stairs Up	Stairs Down	Jogging	ISO Stem	ISO Neck
**M**_**bn**_ **[Nm] in Neck**	24.3	54.4	59.8	***52*.*8***^b^	59.3	69.8	71.2	***81*.*5***^a^	107.1	(56.7)	131.7
**ΔM**_**bn**_ **[%]**	-0.7	1.2	0.5	1.5^a^	0.5	0.8	0.0^b^	0.6	0.3	---	---
**M**_**ts**_**[Nm] in Stem**	15.5	22.9	19.3	15.6	24.8	***11*.*7***^b^	***47*.*0***^a^	32.4	57.4	16.0	(37.0)
**ΔM**_**ts**_ **[%]**	***1*.*0***	***-1*.*8***^b^	2.9	1.7	0.8	***7*.*2***^a^	0.9	-0.3	-0.4	---	---
**M**_**bs**_ **[Nm] in Stem**	36.8	57.0	46.4	***36*.*8***^b^	65.9	55.4	***113*.*1***^a^	90.2	149.1	69.6	(161.5)
**ΔM**_**bs**_ **[%]**	2.7	***-3*.*3***^a^	5.2	***5*.*5***^a^	0.6	-1.0	0.0	0.2	0.2	---	---

^a^/^b^ = ***highest/lowest*** absolute values during activities except cycling and jogging.

The ISO *neck* force produced a *bending* moment M_bn_ of 131.7 Nm in the neck; this was higher than any in vivo value, including that from jogging (Table 7). Regarding the local moments in the implant neck and stem (Table 7), the most strenuous in vivo activities were ascending or descending stairs (jogging excluded).

The bending moment M_bs_ in the stem was nearly the same for the ISO *stem* test (69.6 Nm, Table 7) and for walking (65.9 Nm), although F_res_ was lower for the ISO stem test (2300N) than for walking (2880N, Table 5). When changing the orientation of the implant relative to the femur by modifying all three angles α_x_, α_y_ and α_z_ (Fig 1) by ±3°, the largest change of M_bs_ during walking, caused by any combined angle variations, was ±40%.

### Influence of friction moments on loads in neck and stem

If the contact forces were considered without friction, the highest bending moment M_bn_ in the *neck* was 81.5 Nm when going down a staircase (Table 7, cycling and jogging excluded). If the friction moments were added to the analysis, M_bn_ rose by a maximum of +1.5%. Without friction moments, the highest torque M_ts_ around the implant *stem* was 47.0 Nm during stair climbing. Friction increased M_ts_ by 7.2% during stance, but changes for the other activities only reached 2.9%. If only the contact forces acted, M_bs_ was highest at 113.1 Nm when ascending stairs (jogging excluded). Changes of M_bs_ due to friction varied between +5.5% and -3.3%.

### Data for internet download

The data obtained from this study and additional information can be downloaded as numerical files and graphics from the public database www.Orthoload.com. The AVER75, HIGH100, individual load courses, and video records of the individual subjects during all investigated activities are available. If required, the numerical data can be used directly as input for analytical or experimental investigations.

## Discussion

### Comparison of data with our previous publication

The data presented were based on a much larger group of ten subjects and included younger and more physically active individuals compared with our previous study [8]. This earlier reference was based on in vivo measured contact forces from only four older and less active subjects. Furthermore, in the previous study only the contact forces but not the moments could be measured. For all activities (cycling and jogging excluded), the peak values of F_res_ in the AVER75 data were larger in the current than in the previous study. The differences were between 11% and 25% and even 60% for knee bend. This 60% increase was probably caused by the higher hip flexion angles in the new group. The differences in the remaining activities may be explained in part by the faster execution of the exercises. Because the previous study included only four subjects, statistical significances of the differences could not be tested.

### Extreme loads, not included in the standardized data

In the previous study [8] extremely ‘high forces’ up to 11000 N during stumbling were reported. Because such uncontrolled movements could not be investigated systematically, even higher occasional contact forces cannot be excluded. Loads of 11000 N are 2.3 times higher than the HIGH100 loads during jogging and 3.8 times above those during walking, as reported here. Because the static strength of metal implant materials is 2 to 3 times higher than the fatigue strength [6], these rare extreme forces could endanger the neck and stem of the implant. Therefore, it is suggested that static tests or tests with low cycle numbers for such extreme forces should be performed *in addition* to the fatigue tests.

The standardized HIGH100 loads describe high load levels in subjects with a BW of 100 kg. It had already been mentioned that these loads should be increased by 25% to encompass the high BW of 60–69 year old male patients in the United States. It is not possible to define loads for extremely obese subjects, during accidents or loads caused by frequent and excessive activities, such as fast running or playing tennis. For such conditions, any upper limitation and standardisation would be arbitrary. Such loading conditions will always cause an increased risk of implant failure that cannot be quantified.

### Load transformations for endurance tests of the stem

The presented forces and moments all refer to the coordinate system x, y, z of the femur (Fig 1). For endurance tests of the femoral component, however, these loads are preferably applied in the x’, y’, z’ system of the implant. For such a load transformation, the three orientation angles, α_z_, α_y_, and α_x_, of the implant have to be defined and the HIGH100 force and moment components must be re-calculated according to Eqs 1 to 3. For the implant orientation of our *average* subject (Table 4), the transformation matrix from Eq 4 can be applied.

### Suggestion on approaches to realistically test hip implants

The HIGH100 loads are based on data from only ten subjects. Due to inter-individual variations, the loads would probably increase if data from more subjects were included. This is illustrated by the atypically high AVER75 forces in one subject (H5L) during the double-legged activities (Fig 3). It is likely that even higher loads than those reported here among the general population of joint replacement patients are possible. Additionally, it is common technical practice to perform fatigue tests at a load level that is raised by a safety factor above its expected maximum. This would suggest to test the implants at even higher load levels than reported here.

Most endurance tests of the implant stem are currently performed by applying uni-directional sinusoidal forces, as defined in the ISO standards. If this practice is to be continued, the direction and amplitude of the test force should be adapted to realistic HIGH100 forces. One *possibility* could be to combine the highest activity amplitude of F_res_ (3875 N in stair descent) with the *average* force directions α_front_ and α_sagit_ from the most frequent activity (walking) and the most demanding activity (ascending/descending stairs), which would be 17.5° and 13.5°, respectively (Table 5). If only walking should be simulated, a maximum force of F_res_ = 2880 N would have to act at α_front_ = 17° and α_sagit_ = 11°.

A more realistic test method would be to apply a combination of HIGH100 *peak* forces from different activities with sinusoidal time courses. Such a test would either require a 3D loading apparatus or multiple re-arrangements of the stem. Based on the duration and frequency of daily activities in total hip replacement patients [17], the following activity numbers were proposed [8]: 1000 (Walking) + 55 (StairsUp) + 55 (StairsDown) + 25 (StandUp) + 25 (SitDown) + 50 (Stance). For a total cycle number of 10^7^, these numbers must be multiplied by 10^4^. If the testing machine enabled loading of the femoral component under varying directions, the real HIGH100 load-time course during different activities could be applied instead of forces with constant directions.

The data from walking (Fig 8, small dots) and the other activities (Table 5) indicate that the *individual* force directions differ considerably from the *average* directions of either AVER75 or HIGH100 forces. These individual variations are disregarded when applying unidirectional forces or a combination of time-dependent HIGH100 forces, as proposed for a simplified test. Thus, it may be of interest to test endurance at a defined location at the neck or stem under the worst possible loading conditions. For such a scenario, the *absolute* maxima of each of the six *internal* force and moment components at this location can be determined from all *individual* loads and for a special collection of activities. Such a collection may include the proposed combination of the six most demanding activities or just walking, for example. Because all three force components at the head act in one axis direction and don’t change their signs (Figs 4 and 8), one specific unidirectional force at the head can be calculated, which *exactly* generates three of these six maximum *internal* load components. In most applications, this will be the three *moment* components, as they have the greatest impact on the local stresses. A test force derived in this manner would be best suited to mimic the worst stress condition at the selected location. A ‘worst condition at a given location’ test force could be especially valuable for testing implants of different sizes or the endurance of the taper in modular implants. We plan to support such developments by creating a dedicated, freely available computer program for that purpose in the future.

### Analysing the mechanical situation around the implant

The ISO stem test assumes a totally resorbed proximal femur. A different situation exists if the femoral component is proximally well fixed and the torsional stem stability or bone remodelling around the stem have to be tested or analysed. Then the muscle forces acting at the upper femur must also be taken into account as they widely influence bone deformation and stresses and strains in the implant and interface [27, 28]. Knowledge about these forces during various activities, however, requires verified musculoskeletal analyses [29–31]. The data presented here can be used to improve predictive power and accuracy of such simulation models.

Friction moments are important for the stability of the cup in the acetabulum, especially for cementless fixations. However, the stresses in the bone surrounding the cup additionally depend on the magnitude and direction of the contact forces relative to the cup. Higher contact forces are likely to increase the torsional strength of the cup fixation due to a clamping effect. Although a detailed analysis of the acetabular cup loading was beyond the scope of the current paper, gait data were captured in all subjects to determine hip joint kinematics and to allow the necessary transformation of contact forces and moments from the femur to the acetabulum. Future publications will focus on the analyses of the hip kinematics and the orientation of the cup loads because this information is needed to test wear and stability of the cup.

If quasi-static problems are investigated, e.g., the *sudden* failure at a porous stem interface during the first postoperative weeks, *extreme* joint contact forces must be considered. Such excessive loads may act during stumbling, for example. During such events, when substantial levels of muscle co-contraction prevail in an attempt to stabilise the joint, joint contact forces of up 870% BW were measured [7], corresponding to 11,000 N in a 100 kg subject with high load levels [8]. Such extreme forces were observed only by chance and never in planned laboratory experiments or by maximal arbitrary muscle activities. However, because the load directions during stumbling were found to be similar to those during walking, it seems reasonable to approximate such loading conditions by applying the HIGH100 force directions from walking but increasing the force magnitude to 11,000 N.

### Internal moments in neck and stem when applying ISO or HIGH100 forces

The ISO stem force generated torque in the *stem* that was smaller than that during most in vivo activities, including walking (Table 7). The bending moment by the ISO stem force was just sufficient to mimic the conditions during walking but was 38% smaller than that observed when ascending stairs. Amplitude as well as direction of the ISO stem force are therefore insufficient for testing the endurance of the stem. The use of the new in vivo load data presented here should permit a better approximation of the mechanical in vivo situation of the stem.

When modifying all three implant orientations relative to the femur at the same time by ±3°, the bending moment M_bs_ changed by up to ±40% during walking. This shows that the stresses in a proximally resorbed implant not only depend on the magnitude of the contact force but also on a) the curvature of the femur in two planes, b) the anteversion of the implant and c) the chosen location on the stem.

Fractures of the stem are rare. A proximally resorbed femur is more likely to cause a periprosthetic fracture (0.47% risk for uncemented stems) than a stem failure [32]. This clinical observation and the susceptibility of the moments to the factors a) to c) call into question the relevance of the stem tests.

### Influence of friction moments on loads in neck and stem

Although the internal moments in neck and stem are influenced by the presence of additional friction moments, their influence is small. The largest increase of any internal moment due to friction was 7.2%. For some activities, friction even slightly reduced the local moments. Compared to the influence of the contact forces, the impact of friction on the *internal* implant loads is small. Given the complexity of modelling friction in a fatigue test, the effect of friction could be alternatively modelled by increasing the contact force by approximately 7%.

### Moments at the acetabular cup

The highest HIGH100 friction moments M_res_ reached values of up to 2.8 Nm during standing up, descending stairs and knee bending. Our internal data collection contains occasionally measured moments up to 6.1 Nm (not published). Moreover, three months after surgery, the friction moments were between 13% and 72% (47% average in 10 subjects) higher than they were a year after the operation [20] and exhibited substantial individual variability. Therefore, peak in vivo moments can exceed the reported minimum fixation strength limit for the cup, which lay at 2.2 Nm or 3.3 Nm. Thus, the fixation of cementless cups may be at risk, especially soon after the operation, if the components are implanted with only minor under-reaming in subjects with high load levels, a high body weight or higher than average friction moments. This risk is highest when standing up, descending stairs and during knee bending (Table 5), but the mechanics during other activities are only marginally less strenuous.

The observation that the friction-induced moment components M_y_ and M_z_ were higher during static stance than during nearly all dynamic activities, including jogging, is of special interest. This probably indicates that friction during stance is high because the synovia is squeezed out of the joint by the permanent static load. This worsens the lubrication and relatively small forces can then generate high moments.
